# Exploring contractile protein mechanisms and target medications for cardiomyopathic patients with diastolic dysfunction

**DOI:** 10.1002/pdi3.60

**Published:** 2024-03-25

**Authors:** Dustin Gerber, Junjun Quan, Bo Pan, Xupei Huang, Jie Tian

**Affiliations:** ^1^ Department of Biomedical Science Charlie E. Schmidt College of Medicine Florida Atlantic University Boca Raton Florida USA; ^2^ Department of Cardiology Children's Hospital of Chongqing Medical University Chongqing China; ^3^ National Clinical Research Center for Child Health and Disorders Ministry of Education Key Laboratory of Child Development and Disorders Chongqing China

**Keywords:** cardiomyopathy, diastolic dysfunction, EGCG, green tea extract, mavacamten

## Abstract

Genetic defects have been increasingly found in cardiomyopathies, which are often present with mutations in cardiac contractile proteins. These congenital defects involve numerous intracellular pathways and share several critical clinical features, such as systolic or diastolic dysfunction fostering the various cardiomyopathic phenotypes. Hypertrophic cardiomyopathy and restrictive cardiomyopathy (RCM) share a common pathological feature, that is, diastolic dysfunction. Studies have shown that mutations of contractile proteins, especially myosin heavy chain and troponin, are tightly associated with diastolic dysfunction in patients with Cardiomyopathies (CMs), including pediatric patients with CM. Therapeutics, including green tea extract (epigallocatechin gallate) and mavacamten, interact directly with these contractile proteins and have shown promising results. This article will review recent and contemporary research on diastolic dysfunction in CMs, especially hypertrophic cardiomyopathy and RCM, which include their target proteins, mechanisms, clinical diagnosis, and potential therapies.

## 1 INTRODUCTION

Cardiomyopathies (CMs) are heterogenous diseases that commonly impact the heart muscle.^1^ There are three universal classifications of cardiomyopathy based on representative morphology and pathophysiology: dilated cardiomyopathy (DCM), hypertrophic cardiomyopathy (HCM), and restrictive cardiomyopathy (RCM). Hypertrophic cardiomyopathy and RCM share a similar pathological feature of cardiac diastolic dysfunction (CDD). Cardiac diastolic dysfunction refers to impaired relaxation and an abnormality in heart filling during diastole while left ventricular (LV) systolic function is preserved.^2^, ^3^ CDD is also a precondition of heart failure with preserved ejection fraction (HFpEF), which has a prevalence from 1.1% to 5.5% in the general population.^4^

At past, most patients with diastolic dysfunction are treated with medications such as betablockers, diuretics, etc. For their clinical symptoms and later develop heart failure because these medications are not targeted to the etiology mechanisms or molecules.^5^ As a result, there was no effective medication that worked specifically on etiological molecules (proteins) in diseased myocardial cells. Increasingly now, genetic defects have been found in diastolic cardiomyopathies, which are often present with mutations in cardiac contractile proteins, especially myosin heavy chain (MHC) and troponin therapies. In recent years, specific medications targeting this mechanism have achieved positive results. Therefore, this article reviews the recent studies, including our experimental data on diastolic dysfunction in CMs, namely HCM and RCM caused by contractile protein mutations.

## 2 CHARACTERISTICS OF CARDIOMYOPATHIES AND CARDIAC DIASTOLIC DYSFUNCTION

Cardiomyopathies are considered to represent diseases that primarily affect cardiac muscle. On the ground of their morphology and pathophysiology, cardiomyopathy is classified into three major types: HCM, DCM, and RCM. Hypertrophic cardiomyopathy and DCM are characterized, as their name indicates, a hypertrophic heart or a dilated ventricle. In contrast, RCM manifests itself as the restricted amount of blood that can fill the heart because it is abnormally stiff due to unknown etiology. The clinical features of RCM include bi‐atrial dilation and normal LV internal dimension characterized by echocardiography.

Pediatric cardiomyopathies are diseases in children with an annual incidence of 0.7–1.2 per 100,000.^6^ DCM and HCM are the most common, whereas RCM and other cardiomyopathies occur infrequently. Hypertrophic cardiomyopathy is the second most common pediatric cardiomyopathy, accounting for about 35%–50% of patients with an annual incidence of 0.47 per 100,000 children,^6^, ^7^ and is described as a hypertrophic heart disease with increased cardiac wall thickness. Restrictive cardiomyopathy, however, is a rare disease in children, only accounting for less than 5% of all pediatric cardiomyopathy.^6^ Specifically, RCM is a cardiomyopathy that shows ventricular diastolic dysfunction with preserved systolic function, bi‐atrial dilation, and an absence of ventricular hypertrophy or dilatation. Although RCM is the least common pediatric cardiomyopathy with an incidence of 0.025–0.030 per 100,000 children, it has a very poor prognosis with an approximate death rate of 63% three years after diagnosis.^8^ Occasionally, symptoms of pediatric cardiomyopathy are mistaken as a cold, flu, asthma, or stomachache. Some affected children have no symptoms, while others may experience any of the following symptoms: shortness of breath or rapid breathing, dizziness and fainting, sweating while nursing or taking a bottle, poor weight gain, irritability, and lethargy. Compared to adult patients, chest pain and extreme fatigue are uncommon in pediatric cardiomyopathy patients.

Hypertrophic cardiomyopathy and RCM share a similar pathological feature of diastolic dysfunction. The diastole is part of the cardiac cycle that includes four periods: the isovolumic relaxation period, the rapid filling period, diastasis, and atrial systole. Cardiac diastolic dysfunction is a precondition HFpEF. Heart failure with preserved ejection fraction is a clinical syndrome mainly associated with decreased active ventricular relaxation during the early diastolic period and increased passive myocardial stiffness during the late diastolic period. The overall prevalence of HFpEF is from 1.1% to 5.5% in the general population and higher in older adults.^4^ CDD can be the consequence of abnormalities during any phase of the diastole.^9^ Though impaired relaxation, high ﬁlling pressure, increased LV operating stiﬀness, mechanical asynchronism, increased peripheral artery stiﬀness, and loss of atrial contraction at higher heart rates may be the features of CDD, its diagnosis can be challenging due to lacking specific symptoms and signs of CDD and mainly depends on echocardiography, along with regular tests of chest radiograph and electrocardiogram. Patients who undergo persistent CDD manifestations might have heart failure related to CDD.

## 3 GENE MUTATIONS IN HYPERTROPHIC CARDIOMYOPATHY AND RESTRICTIVE CARDIOMYOPATHY

There are many causes of cardiomyopathy in children, including some that are not fully understood. Common causes include inheriting the condition from one or both parents, a viral infection, toxins affecting other organs, and metabolic, mitochondrial, or systemic diseases in parts of the body other than the heart. Increasingly, the importance of genetic mutations in the pathogenesis of isolated or syndromic pediatric cardiomyopathies is becoming apparent.

Many studies have demonstrated that HCM is one of the most frequently occurring inherited cardiac disorders. Furthermore, more than 50% of human mutations of HCM occur in β‐myosin heavy chain (*MYH7*) and myosin binding protein C. Additional genes implicated in HCM include troponin T (*TNNT2*), α‐tropomyosin (*TPMI*), myosin regulatory light chain (*MYL2*), myosin essential light chain (*MYL3*), and troponin I (*TNNI3*).^10^, ^11^, ^12^, ^13^ Recently, several studies have demonstrated that HCM is one of the most frequently occurring inherited cardiac disorders, and many mutations in genes for sarcomeric proteins, including cTnI, cTnT, cTnC, and α‐tropomyosin (Tm), have been found to cause HCM.^10^, ^12^, ^13^ Troponin I can be classiﬁed into four functional regions: the N‐terminal TnC‐binding region, the TnT binding region, the inhibitory regions, and the C‐terminal TnC‐binding region. In 1997, five cTnI mutations, R145G, R145Q, R162W, G203S, and K206Q, were firstly reported for being associated with human HCM.^14^ Since then, dozens of cTnI mutations have also been reported. cTnT, as another subunit of troponin complex, was for the first time exemplified for being associated with HCM in 1994.^15^ Recently, cTnC, a Ca^2+^‐binding regulatory protein, has been linked to HCM. Several studies have shown that L29Q mutation in cTnC could abolish the effect of force‐generating cross‐bridges on the Ca^2+^ sensitivity of the structural changes, which could contribute to the pathogenesis of HCM.^16^, ^17^ Except for troponin mutations, the variants in thin filament protein α‐tropomyosin were also identified in HCM patients.^18^ Mutated gene in *myosin‐binding protein (MYBPC3)*, encoding cardiac myosin‐binding protein C of thick ﬁlaments, has been linked to HCM.^19^

In 2003, cardiac troponin I (cTnI) C‐terminal mutations were firstly reported for being associated with human RCM. At that time, six cTnI mutations (L144Q, R145W, A171T, K178E, D190G, and R192H) had been found to be associated with RCM. Among them, the two mutations K178E and R192H had the worst clinical phenotype.^20^ Recent studies have shown that sarcomeric mutations in troponin I (*TNNI3*), myosin‐binding protein (*MYBPC3*), β‐myosin heavy chain (*MYH7*), myosin light chain genes (*MYL3*), troponin T (*TNNT2*),α‐cardiac actin, troponin C (*TNNC1*), tropomyosin 1 and myosin light chain (*MYL3* and *MYL2*), and nonsarcomeric mutations in filamin‐C and desmin *(DES)* are contributed to the pathogenesis of RCM.^21^, ^22^, ^23^, ^24^, ^25^, ^26^ Among these mutations, *TNNI3* mutation is a very common pathogenic cause for RCM.^20^, ^27^, ^28^, ^29^ To date, most of the RCM mutations in cTnI have not been incorporated into transgenic models, though they have been characterized in functional in vitro studies.^30^, ^31^, ^32^, ^33^, ^34^, ^35^

## 4 MECHANISMS UNDERLYING DIASTOLIC DYSFUNCTION AND CARDIOMYOPATHIES

Cardiac diastolic dysfunction can result from various pathological conditions, such as hypertension, diabetes, cardiac fibrosis, atherosclerosis, and cardiac contractile protein malfunction. Here we focus on diastolic dysfunction in cardiomyopathies, which is caused by contractile protein mutations and malfunction in myocardial cells. In myofibrils, myosin is a motor protein that causes crossbridge formation when myosin attaches to thin filament actin. The ATPase in myosin heads is critical, which hydrolyzes adenosine triphosphate (ATP) into adenosine diphosphate and Pi providing the energy for muscle sliding. ATPase and crossbridge formation dynamics determine the force production and the contraction and relaxation cycles.

Conversely, myocardial thin filament proteins such as troponin complex (troponin C, T, and I) are equally important in regulation of cardiac muscle contraction and relaxation. In the systole, Ca^2+^ binds with troponin C (TnC), which causes a conformational change of troponin and tropomyosin, leaving the space for crossbridge formation, that is, attachment between myosins and actins. Previously we paid more attention to calcium concentration since an increased calcium concentration means an increased contractility (positive inotropy), and a decreased calcium concentration means a reduced contractility (negative inotropy). However, the myofibril sensitivity to Ca^2+^ (calcium sensitivity) in cardiac myocytes has attracted more attention recently. Studies have shown that calcium sensitivity is critical in regulating cardiac muscle contraction and relaxation. In the following, we will discuss the mechanisms of myosin and troponin caused by diastolic dysfunction in cardiomyopathies.

### 4.1 Myosin in diastolic dysfunction and cardiomyopathies

The definition of HCM is morphologically a significantly enlarged LV wall with a decreased LV chamber. Functionally, HCM is described traditionally as increased contractility (hypercontractility). Hypercontractility is often observed in HCM patients with mutations in β‐cardiac myosin.^36^ Therefore, HCM, especially HCM caused by myosin mutations, was hypothesized to increase myosin activity at the protein level, resulting in increased force production at the sarcomere and cellular levels, propagating to the whole heart.^36^, ^37^

Biochemical and biophysical measurements characterize myosin protein activity. The activity of actively cycling myosin and interacting with actin is characterized by the rate of ATP turnover, the rate of detachment from actin, force production, step size, and actin‐sliding velocity.^37^ Recent studies have demonstrated that myosin exists in three different functional states in the muscle filaments. The first is the active cycling state of myosin, which involves actin‐activated ATP hydrolysis and a rapid ATP turnover time of <1 s. The second state, often called the disordered relaxed (DRX) state, is the ATP turnover state of myosin in the absence of actin, which has a prolonged ATP turnover time of <30 s. The third, or the super relaxed state (SRX), is the state that has even more prolonged ATP turnover times of more than 100 s.^38^ The population of the SRX state is measured kinetically by following the release of fluorescent ATP hydrolysis products from myosin, and the right population of the super‐relaxed state ensures muscle sarcomeres in a proper contraction and relaxation cycle. The SRX state of myosin is only recently reported in cardiac muscles.^38^ It is characterized by a subpopulation of myosin heads with a highly inhibited rate of ATP turnover.^38^ Myosin heads in the SRX state are bound to each other along the thick filament core producing a highly ordered arrangement. The structural basis of super‐relaxation is usually taken to be a motif formed by myosin in which the two heads interact with each other and with the proximal tail creating an interacting‐heads motif, which switches the heads off. Studies have shown that the ability of myosin to form the folded‐back interacting‐heads motif structure is critical for regulating myosin activity, and many HCM‐causing mutations appear to disrupt the ability of myosin to enter the SRX state^37^, ^39^, ^40^, ^41^, ^42^ In other words, myosin mutation caused HCM results in a large population of myosin in HCM heart shifting from the SRX state to the disordered relaxed state (DRX), the consequence of which is increased myosin–actin interaction, more possible crossbridge formation, and greater force production in the diseased heart.

### 4.2 Troponin in diastolic dysfunction and cardiomyopathies

Troponin plays a central role in the calcium‐regulation of muscle contraction: troponin is the sole calcium‐binding component of thin filaments (actin‐tropomyosin‐troponin complex) of striated muscles. Thin filaments without troponin support contraction irrespective of calcium concentration.^43^ Regulatory troponin is a complex consisting of three subunits: Ca^2+^‐binding troponin C (TnC), tropomyosin‐binding TnT, and inhibitory troponin I (TnI). During excitation–contraction coupling in myocardial cells, Ca binds with cTnC causing a conformational change in the whole troponin complex, which in turn moves tropomyosin away from the space between myosin and actin molecules. Then myosin interacts with the actin resulting in crossbridge formation and muscle contraction. For the first time, our laboratory generated a cTnI gene knockout mouse model in 1999.^44^ Characterization of the cTnI knockout mice (−/−) has demonstrated that cTnI is critical in the regulation of myofibril contraction and relaxation. Without cTnI, cardiac cells produce more calcium‐unrelated force and have a shortened sarcomere even in relaxing states.^44^ Our studies confirm that cTnI can “inhibit” the myosin/actin interaction, that is, crossbridge formation in myocardial cells, which ensures a proper relaxation time in cardiac muscles. In the absence or deficiency of cTnI, “over‐contraction” or impaired relaxation occurs, resulting in diastolic dysfunction and even diastolic heart failure in those mice.^44^, ^45^

Recently, several laboratories including ours have demonstrated that cardiac myofibril hypersensitivity to Ca^2+^ is one of the key factors resulting in impaired relaxation in myocardial cells.^17^, ^46^, ^47^, ^48^ Based on 1 of the 6 gene mutations with the worst clinical phenotype for RCM, we have generated gain‐of‐function transgenic mice modeling mutations in human cTnI C‐terminus, cTnI R192H, by cardiac specific expression of the mutated protein in mouse heart (cTnI^193His^ in mouse sequence). Characterization of this mouse model demonstrated that cTnI^193His^ mice develop diastolic dysfunction about two months after birth, leading to RCM and diastolic heart failure causing early death.^49^, ^50^ Cellular and molecular studies have demonstrated that increased myofibril sensitivity to Ca^2+^ in this mouse heart is a critical feature that impaired these myocardial cells' with cTnI R193H mutation.^5^, ^6^, ^48^ Morphologically, cTnI R193H mice have a phenotype like that in human RCM patients carrying the same mutation characterized by enlarged atria and restricted ventricles.^49^ Furthermore, we have demonstrated that impaired relaxation is a main manifestation in the RCM cTnI transgenic mice^49^ and cTnI mutation caused myofibril Ca^2+^ hypersensitivity is a key factor resulting in a delayed calcium drop‐off from the myofilaments and a prolonged relaxation time.^51^, ^52^

The C‐terminal of troponin I, amino acids 184–210 in human cTnI, is the most conserved domain of the molecule and interacts with tropomyosin in a calcium‐regulated manner, suggesting it is a functional role. In addition, the remaining region of cTnI, amino acids 190–210, has been shown to play a vital role in stabilizing tropomyosin in the actin filament upon Ca^2+^ activation.^46^ The cTnI molecular integrity is essential for proper conformation of the troponin complex in the myoﬁlament and the inhibition of actomyosin ATPase activity. In vivo and in vitro studies have demonstrated that cTnI mutations in the remaining segment can cause myofibril Ca^2+^ hypersensitivity and subsequent impaired relaxation, which is the primary manifestation of RCM.^5^, ^46^, ^53^

In addition, we also generated another transgenic mouse carrying the RCM cTnI K178E mutation (cTnI K179E in mouse sequence), which was marked by similar hemodynamic and morphological characteristics to the cTnI^193His^ RCM animals.^54^ Additionally, similar to the results of our studies, cTnI R145W transgenic mice created by other laboratory has shown that the myoﬁbers with R145W mutation have a drastic elevation in Ca^2+^ sensitivity of both force development and ATPase by reducing the interaction between helix‐C of cTnC and cTnI.^53^, ^55^ Recently, more and more mutations in cTnI, such as R204H and R204C, have been verified to be associated with increased myoﬁbril Ca^2+^ sensitivity and subsequent impaired relaxation.^56^

## 5 TREATMENT OF DIASTOLIC DYSFUNCTION AND CARDIOMYOPATHIES

**FIGURE 1:**
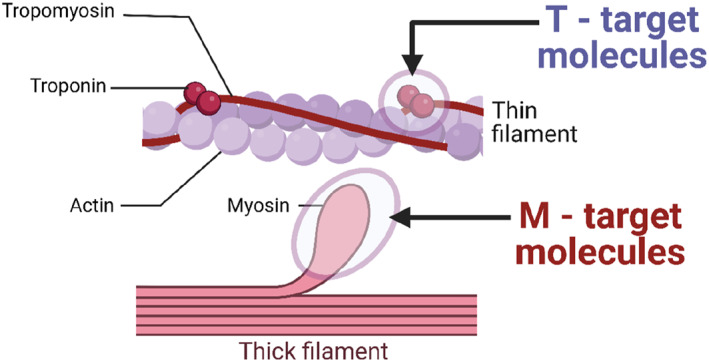
Cardiac contractile proteins myosin heavy chain (MHC) and troponin are target proteins in Hypertrophic cardiomyopathy (HCM)‐and restrictive cardiomyopathy (RCM)‐causing mutations. They are the targets as well in exploration of myosin modulators (M‐molecules) and troponin modulators (T‐molecules) for the treatment of diastolic dysfunction and cardiomyopathies.

The mechanism studies have targeted two major cardiac contractile proteins, myosin and troponin, associated with impaired relaxation (diastolic dysfunction) in cardiomyopathies, especially in HCM and RCM. The former causes diastolic dysfunction by disrupting the SRX in myocardial filaments that do not affect myofibril sensitivity to Ca^2+^. The latter causes a diastolic dysfunction by increasing myofibril sensitivity to Ca^2+^ and Ca^2+^/cTnC binding affinity (delaying Ca^2+^ drop‐off time from cTnC). The data obtained from the mechanism studies gave us some clues in searching for novel medicines, especially some small chemical molecules, to treat the disorder. One class is myosin modulators (M‐targeting molecules) that targets myosin ATPase and myosin head movement. The other class is troponin modulators (T‐targeting molecules) that target troponin by regulating the Ca^2+^/cTnC binding and myofibril sensitivity to Ca^2+^. Figure 1 illustrates the M‐targeting molecules and T‐targeting molecules working on different sites of the cardiac myofilaments (Figure 1).

Animal models exhibiting distinctive pathological features analogous to human heart diseases and heart failure have been generated to identify the fundamental causes of heart diseases. Using animal models makes it possible to test preventive or reparative therapies that reduce heart disease morbidity and mortality.^57^ Most animal models of human cardiomyopathies and heart failure mimic systolic dysfunction and systolic heart failure caused by aortic ligation or injection of toxic chemicals in human patients. The animal models of diastolic dysfunction or diastolic heart failure are limited.^57^, ^58^

Using a transgenic mouse model of cardiomyopathy with diastolic dysfunction, we have elucidated the relationship between Ca^2+^ hypersensitivity caused by myofibril protein mutations and the following diastolic dysfunction in the heart.^4^, ^5^ There is a great need to develop or find small molecules and chemical Ca^2+^ desensitizers that can be used to alter myofibril sensitivity for Ca^2+^ and myofilament sliding kinetics. A significant concern about biological agents is their non‐toxicity and good bioavailability, but only few Ca^2+^ desensitizers possess such qualities. The catechin, (−)‐epigallocatechin‐3‐gallate (EGCG), has been newly found to contain Ca^2+^ desensitizing abilities via its interaction with cTnC.^59^, ^60^ This compound is the most abundant catechin in green tea and is credited for the numerous health benefits attributed to green tea consumption.^61^ EGCG desensitizes myofilament to Ca^2+^ by forming a ternary complex with the C‐terminal domain of troponin C and the anchoring region of cTnI.^60^ The affinity of TnC to Ca^2+^ is reduced, facilitating cardiac relaxation. The ability of EGCG to correct myofilament Ca^2+^ hypersensitivity and diastolic dysfunction has been demonstrated in an HCM mouse model and in our RCM cTnI^193His^ mice, confirming the therapeutic potential of that compound for diastolic dysfunction.^51^, ^52^, ^62^, ^63^

Experiments in vivo have indicated that catechin EGCG is a calcium desensitizer that interacts with cardiac troponin‐C to reduce myoﬁlament Ca^2+^ hypersensitivity.^60^ A recent study reported that Ca^2+^ sensitivity of the myofilament containing the cTnI variant K206I was significantly increased, and, when treated with EGCG, was restored to wild‐type level in ATPase and force measurements, suggesting that EGCG could normalize the enhanced Ca^2+^ sensitivity of myofilaments caused by associated mutation of human cTnI.^64^ Based on this specialty, catechin possesses the therapeutic potential to improve impaired heart relaxation and diastolic function in RCM or HCM.^63^, ^64^ Another study from our laboratory further indicated that EGCG was effective in correcting CDD in RCM mice mainly by improving ventricular compliance and reducing the internal muscle rigidity caused by myofibril hypersensitivity to calcium.^65^

Some studies also showed that EGCG could affect Ca^2+^ sensitivity in thin ﬁlaments containing cTnI mutations associated with HCM in a phosphorylation dependent way and restore the coupled relationship between Ca^2+^‐sensitivity and troponin I phosphorylation in mutant thin ﬁlaments to normal.^12^, ^66^

The *MYBPC3‐*associated HCM mice exhibit, in addition to LV hypertrophy and decreased fractional shortening, increased Ca^2+^ sensitivity and diastolic dysfunction.^19^ The ﬁndings in these studies support that interventions decreasing myoﬁlament Ca^2+^ sensitivity could reverse the phenotype of HCM and have a therapeutic value.^3^, ^67^ In vitro, 1.8 μM EGCG increased diastolic Ca^2+^, accelerated Ca^2+^ transient kinetics, and shortened relaxation time in isolated cardiomyocytes from *MYBPC3‐*associated HCM mice but had no effect on diastolic sarcomere length.^47^ Furthermore, 30 μM of EGCG decreased myoﬁlament Ca^2+^ sensitivity to a greater extent in *MYBPC3*‐targeted knock in than in wild type skinned ventricular trabeculae.^47^ Another study in cats showed that HCM cats with *MYBPC3* R820W variant had a higher Ca^2+^‐sensitivity than non‐HCM cats, and blunted modulation of Ca^2+^‐sensitivity by TnI phosphorylation in HCM cats could be restored by 100 μM EGCG.^48^ These studies suggest that myofibril Ca^2+^ hypersensitivity, which results from mutations in thin and thick myoﬁlaments, play a similar role in the pathogenesis of HCM and RCM and can be reversed by green tea catechin EGCG.

Due to the properties of EGCG, we have carried out a study on pediatric patients with RCM or HCM. In the study, the RCM and HCM cardiomyopathy patients with diastolic dysfunction were treated with a daily intake of EGCG for 12 months. The study has demonstrated the beneficial effect of daily administration of EGCG in cardiomyopathy patients with diastolic dysfunction.^68^ Significant increases of left ventricular end diastolic volume and stroke volume were observed by echocardiography in the patients. In addition, an improvement of the E/A ratio was also detected in these patients.^68^ Furthermore, no adverse side effects were observed in all patients treated with EGCG during the whole study. Interestingly, RCM patients with a mutation of *TNNI3* had a better effect on cardiac function, indicated by echocardiographic measurements and the brain natriuretic peptide level.^68^ Consistent with our study, the other group has also illustrated that cTnI mutation‐induced RCM is the most common type of pediatric RCM and the application of EGCG is effective in improving diastolic function and reducing clinical symptoms in RCM patients.^69^ In that study, the New York Heart Association functional classification in survived patients was improved from Class III to Class II and mean E/E′ was decreased.^69^ These studies indicated that beneficial effects of EGCG in cardiomyopathy patients with diastolic dysfunction were observed; however, further studies are needed by carrying out more randomized, multinational, and double‐blind trials, including more patients to confirm the therapeutic effects of EGCG on diastolic dysfunction in patients with cardiomyopathy.

Although cardioprotective agents such as β‐blockers or Ca^2+^ antagonists have been used to treat HCM, there is no reliable evidence that these drugs could prevent HCM patients from sudden death. Despite well tolerations, newer the Food and Drug Administration (FDA) approved medications including angiotensin receptor‐neprilysin inhibitors and sodium‐glucose cotransporter‐2 (SGLT‐2) inhibitors only manage the symptoms of HFpEF.^70^, ^71^ Moreover, no effective pharmacotherapy is developed because the underlying mechanisms are still unclear.

Studies have shown that most HCMs, especially the HCM‐causing mutations in beta‐MHC, manifest a “hypercontractility” feature with a damaged relaxation in the heart. Hypercontractility results from an increase in the total number of myosin heads interacting with actin during systole due to a weakening in myosin's S1‐S2 intradomain interaction.^72^ In other words, hypercontractility is caused by too many myosin‐actin interactions, that is, crossbridge formation in HCM myocardial cells. From this thinking, a strategy to directly target the myosin motor has been explored to treat patients with HCM by decreasing the abnormal contractility and reducing the myosin ATPase rate in HCM myocardial cells.^73^, ^74^ A high‐throughput screen performed by MyoKardia (a biotech company located in South San Francisco, CA) identified a molecule MYK‐461 as a compound that decreases myosin ATPase activity by acting as an allosteric modulator to stabilize the autoinhibited state of cardiac myosin.^74^ MYK‐461, later named mavacamten, is a small myosin‐binding molecule that can decrease both myosin ATPase activity and the transition of sarcomere cross‐bridges from their weakly to strongly bound conformation in myocardium expressing HCM‐causing mutations in MHC.^75^ Mavacamten is effective not only in animal models with HCM‐causing mutations in beta‐MHC, but it also has beneficial effects on in vivo cardiac function in cardiac myosin‐binding protein‐C (cMyBPC)‐ related HCM by slowing in crossbridge behavior and attenuating hyperactivity in cMyBPC knockout myocardial cells.^76^

A clinical study to evaluate mavacamten (MYK‐461) in adults with symptomatic obstructive hypertrophic cardiomyopathy (EXPLORER‐HCM) started in 2018 with an enrollment of over 250 adult patients (over 18 years old). The study was completed in 2020.^77^, ^78^ The study group reported their results in 2022 that mavacamten was superior to placebo at improving exercise capacity and health status.^79^ Additionally, a phase II clinical study to evaluate MYK‐461b in adults with symptomatic non‐obstructive hypertrophic cardiomyopathy (MAVERICK‐HCM) was conducted during 2018 and ended in 2020. A total of 59 patients (over the age of 18) enrolled in MAVERICK‐HCM. Upon completion of the clinical trial, researchers reported in 2020 that patients with advanced disease progression were the most responsive to mavacamten and well tolerated in most patients.^79^ In April 2022, the clinical use of this medicine was approved by the FDA. Recent application of mavacamten in Chinese patients with HCM reported a positive outcome.^80^, ^81^ However, this medicine is available only under a restricted distribution program called Camzyos™ Risk Evaluation and Mitigation Strategy Program. Since appropriate studies have not been performed on the relationship of age to the effects of mavacamten in the pediatric population, the use of this medicine for pediatric patients with HCM is currently restricted.

## 6 CONCLUSION

Cardiomyopathy is a common cardiac disease in children. Mutations in contractile proteins are tightly related to inherited cardiomyopathies. Hypertrophic cardiomyopathy and RCM, sharing a common pathological feature of diastolic dysfunction, are associated with myosin and troponin mutations. Myosin motor protein and troponin regulatory protein are target molecules in cardiac myofilaments that can cause either myosin disordered relaxing state or the alteration of myofibril sensitivity to Ca^2+^ in HCM or RCM myocardial cells, which are the potential factors resulting in “hypercontractility” or impaired relaxation in cardiomyopathic hearts. Modulators for myosin and troponin, especially the small chemical modulators, reduce hypercontractivity and improve diastolic function in myocardial cells by interacting with either myosin or troponin molecules. Desensitizing green tea extract catechin (EGCG) can reduce myofibril hypersensitivity to Ca^2+^ caused by troponin mutations in myocardial cells. Given its therapeutic effects, EGCG might be a promising drug for CM patients with diastolic dysfunction. In addition, EGCG is a supplemental compound without many side effects and can be used for any age, including pediatric patients. Myosin modulator mavacamten is a recently FDA‐approved medicine that can be used for obstructive HCM due to its effect on crossbridge behavior and hypercontractility in sarcomeres of HCM myocardial cells. However, the use of this medicine in pediatric patients is currently restricted.

## AUTHOR CONTRIBUTION

Dustin Gerber, Junjun Quan and Bo Pan collected data and information and Junjun Quan, Xupei Huang and Jie Tian drafted and reviewed the manuscript. Dustin Gerber prepared the figure.

## CONFLICT OF INTEREST STATEMENT

Jie Tian is the Deputy Editor‐in‐Chief and Xupei Huang is the associate editor of Pediatric Discovery. To minimize bias, they were excluded from all editorial decision‐making related to the acceptance of this article for publication. The remaining authors declare no conﬂict of interest.

## ETHICS STATEMENT

None.

## Data Availability

All data in the text are available and obtained from literatures in Pubmed.
